# Human skin gas profile of individuals with the people allergic to me phenomenon

**DOI:** 10.1038/s41598-023-36615-1

**Published:** 2023-06-10

**Authors:** Yoshika Sekine, Daisuke Oikawa, Michihito Todaka

**Affiliations:** 1 Department of Chemistry, School of Science, Tokai University, Hiratsuka, Kanagawa 259-1292 Japan; 2 AIREX Inc., R&D Laboratory, Hiratsuka, Kanagawa 259-1292 Japan

**Keywords:** Biomarkers, Chemistry

## Abstract

Recent studies have shown that some people claim that their skin gases provoke allergy-like reactions in people in their near vicinity. Such a phenomenon or symptom is called ‘people allergic to me (PATM)’. Although numerous people suffer from PATM, the actual conditions are unknown. The aim of this study was to investigate the characteristics of human skin profiles in patients with PATM by measuring the dermal emission fluxes of 75 skin gases using passive flux sampler and gas chromatography/mass spectrometry. We found common features in the human skin gas profiles of 20 subjects with PATM, with a significant difference from those of 24 non-PATM subjects: greater emissions of petrochemicals, organosulfur compounds, and some aldehydes and lower emissions of aroma compounds and others. The ratio of toluene to benzaldehyde is considered a vital sign that suggests the fundamental of PATM. These findings indicate that PATM is a medically unexplained phenomenon or symptom worthy of further research, which requires an interdisciplinary approach.

## Introduction

Human body odour comprises several volatile compounds emanating from the skin surface, which are known as human skin gases^1–3^. This is usually recognised as a matter of comfort or discomfort for the surrounding people. The possibility of adverse effects of body odours on human health has rarely been investigated. However, a recent study has shown that people claim that their skin gases provoke allergy-like reactions in people in their vicinity, including sneezing, runny nose, cough, itchy eyes, and red eyes^4^. This phenomenon or symptom is called ‘people allergic to me (PATM)’^4,5^. The word PATM is an internet slang generated in multiple community sites on social network services (SNS) related to PATM. Based on the number of comments on SNS, there are potentially thousands of patients with PATM around the world. Most people with PATM experience a sense of victimisation owing to depression, anxiety, suicidal impulses, and other mental disorders. Some of them declare that they exhibit symptoms such as idiopathic chemical intolerance, also known as multiple chemical sensitivity^6,7^, which is associated with low-level chemical exposure. Those who complain of PATM actively discuss their own symptoms, the reactions of people around them, and possible treatments to alleviate the symptoms. However, it seems that various biases are involved in the discussions that occur within communities. Although this is difficult to believe, many people are forced to retire or quit their jobs because of the symptoms of PATM. Unfortunately, the actual conditions are completely unknown because of a lack of scientific reports focusing on PATM. To date, only a few papers have been published: a case report by Kawakami et al.^4^ and letter to the editor by Bian and Ma^5^. Kawakami et al.^4^ measured the levels of human skin gases and microbial species in the nasal cavity of a 35-year-old male subject who claimed to have PATM and found greater dermal emissions of human skin gases, especially toluene (methyl benzene) and other petrochemicals. Bian and Ma^5^ diagnosed an 18-year-old woman with PATM and suggested that the symptoms of PATM are similar to those of a body dysmorphic disorder to a great extent. However, further studies are required to better understand PATM.

Human skin gas is a complex mixture of inorganic and organic volatile compounds released from the skin surface via three emission routes: dermal glands and blood and surface reaction routes^8–11^. When formed in the human body by internal metabolism, they rise to the skin surface with perspiration and sebum secretion (dermal ground route) and/or directly travel from the blood through the dermal layers (blood route)^8,9^. Inhaled exogenous chemicals are also released from the blood route^12^. In addition, skin volatiles are produced from bacterial metabolism or chemical reactions with substrates in sweat/sebum on the skin surface (surface reaction route)^10,11^. Thus, the composition of human skin gas is complex, and the individual skin gas profile is determined by physical, physiological, and psychological conditions, life behaviours, and living environments. Based on its nature, human skin gas has attracted considerable attention as a non-invasive medical biomarker for monitoring conditions such as digestive disorders^8^, diabetes^13^, melanoma^14^, acute poisoning^15^, and severe burns^16^. If PATM is a condition with a common cause, skin gas composition may also have common features that potentially cause allergy-like symptoms in the surrounding population.

To address this issue, the aim of this study was to understand PATM from the viewpoint of the human skin gas profile, which is the source of body odour or scent. Human skin gas profiles were obtained from 20 subjects who claimed to have PATM by measuring dermal emission fluxes (emission rate per area) of 75 volatile compounds, all components for which we have established measurement methods so far, using a passive flux sampler (PFS) coupled with gas chromatography/mass spectrometry (GC/MS)^11,12^. The results were compared with those of 24 subjects without PATM. This is the first study to report that common features exist in the skin gas profiles of patients with PATM.

## Results

### Skin gas profiles of non-PATM and PATM groups

Dermal emission fluxes of 75 skin gases in 44 subjects were measured. The patients were recruited through SNS and word-of-mouth and assigned into two groups based on their declaration: non-PATM and PATM. The non-PATM group comprised 13 male and 11 female participants (age: 18–59, 31 ± 13 years old) who did not claim to have PATM or any other diseases. The PATM group comprised 12 male and 8 female participants (age: 19–53, average 39 ± 12 years old) who suffered from PATM-like phenomena or symptoms without other apparent diseases. Skin gas sampling was conducted by the subjects themselves at the nondominant forearm using PFS for 1 h in their daily life. After sampling, the PFS was sent to the laboratory at Tokai University via a home delivery service, and the collected skin gases were analysed by GC/MS. Table 1 shows the analytical results for the dermal emission fluxes of 75 skin gases. Although the two groups were based on their declarations, significant differences were observed in the cutaneous emissions of several components, resulting in different human skin gas profiles between the non-PATM and PATM groups.

**Table 1:** Emission fluxes of 75 gases emanating from the skin surface of non-PATM and PATM subjects (unit: ng cm^−2^ h^−1^).

Human skin gas	CAS no.	Non-PATM group (*n* = 24)		PATM group (*n* = 20)		Direction	*p* value
		Median	Mean ± SD	Median	Mean ± SD		
1-Propanol	71-23-8	4.1	5.2 ± 2.7	0.52	3.7 ± 7.0		
1-Butanol	71-36-3	1.3	4.3 ± 9.3	2.4	3.1 ± 4.1		
1-Pentanol	71-41-0	2.8	3.6 ± 3.1	0.68	1.8 ± 4.0	>	**
1-Hexanol	111-27-3	2.5	2.8 ± 2.2	0.13	1.3 ± 2.2	>	**
1-Heptanol	111-70-6	2.3	2.7 ± 2.0	0.17	0.89 ± 1.3	>	**
1-Octanol	111-87-5	1.8	3.0 ± 2.8	0.27	0.87 ± 1.4	>	**
1-Nonanol	143-08-8	4.1	4.8 ± 3.2	0.20	0.44 ± 0.84	>	**
1-Decanol	112-30-1	3.4	7.1 ± 11	0.58	2.1 ± 4.9	>	**
**2-Ethyl-1-hexanol**	**104-76-7**	**0.05**	**0.20 ± 0.28**	**1.5**	**2.3 ± 2.9**	** < **	**
Acetaldehyde	75-07-0	1.0	1.4 ± 1.6	0.68	29 ± 106		
Propanal	123-38-6	6.2	5.6 ± 2.4	2.1	7.6 ± 15		
Butanal	123-72-8	1.8	2.3 ± 1.8	1.5	20 ± 48		
**Isovaleraldehyde**	**590-86-3**	**0.74**	**2.1 ± 2.4**	**3.4**	**5.9 ± 7.2**	** < **	*
Pentanal	110-62-3	1.5	1.8 ± 1.1	1.5	4.3 ± 6.3		
**Hexanal**	66-25-1	**1.2**	**1.5 ± 1.2**	**3.5**	**8.2 ± 11**	** < **	*
Heptanal	111-71-7	2.3	3.0 ± 2.9	0.75	1.7 ± 2.3	>	*
Octanal	124-13-0	2.3	2.6 ± 2.6	1.0	2.6 ± 6.1		
Nonanal	124-19-6	1.1	2.7 ± 3.2	1.4	2.7 ± 3.0		
Decanal	112-31-2	0.82	1.8 ± 1.9	0.58	7.5 ± 27		
2-hexenal	505-57-7	1.0	1.5 ± 1.5	0.19	0.32 ± 0.46	>	**
2-nonenal	2463-53-8	0.44	1.3 ± 1.6	0.13	1.9 ± 6.6		
Acetic acid	64-19-7	485	538 ± 292	15	68 ± 118	>	**
Propionic acid	471-25-0	1.3	2.5 ± 2.5	2.9	3.0 ± 2.5		
Butanoic acid	107-92-6	0.77	1.7 ± 1.9	0.67	1.5 ± 2.0		
Isovaleric acid	503-74-2	0.44	0.66 ± 0.66	0.04	0.47 ± 0.69		
Valeric acid	109-52-4	0.88	1.8 ± 2.9	0.28	3.9 ± 7.4		
Hexanoic acid	142-62-1	0.45	1.1 ± 1.6	1.2	3.5 ± 5.4		
Heptanoic acid	111-14-8	0.53	1.4 ± 1.6	0.36	1.1 ± 2.0		
Octanoic acid	124-07-2	0.85	1.4 ± 1.7	1.0	3.3 ± 6.5		
Nonanoic acid	112-05-0	1.0	2.4 ± 2.7	2.3	9.3 ± 18		
Decanoic acid	334-48-5	0.90	1.4 ± 1.7	1.1	5.3 ± 16		
**Acetone**	**67-64-1**	**1.4**	**1.5 ± 0.77**	**17**	**73 ± 186**	** < **	**
2-Butanone	78-93-3	1.3	1.5 ± 1.4	0.18	5.7 ± 17		
2-Pentanone	107-87-9	0.90	1.5 ± 2.1	1.5	2.5 ± 3.1		
2-Hexanone	591-78-6	0.94	1.6 ± 1.6	1.8	2.9 ± 2.9		
2-Heptanone	110-43-0	0.73	1.6 ± 2.4	0.23	0.40 ± 0.53	>	**
2-Octanone	111-13-7	0.82	1.2 ± 1.3	0.11	0.36 ± 0.48	>	**
2-nonanone	821-55-6	0.80	2.3 ± 3.5	0	0.68 ± 1.6	>	**
2-Decanone	693-54-9	2.0	2.5 ± 1.7	0.10	0.27 ± 0.35	>	**
2-Undecanone	112-12-9	1.8	2.5 ± 1.8	0.30	1.6 ± 5.1	>	**
2-Dodecanone	6175-49-1	1.7	2.3 ± 2.0	0.06	0.38 ± 0.62	>	**
2-Tridecanone	593-08-8	1.8	2.4 ± 1.9	0.09	0.32 ± 0.50	>	**
2-Tetradecanone	2345-27-9	2.1	2.5 ± 2.6	0.08	0.71 ± 1.5	>	**
2-Pentadecanone	2345-28-0	2.3	3.4 ± 3.3	0.26	1.9 ± 4.0	>	**
Diacetyl	431-03-8	1.2	2.4 ± 3.1	1.6	3.4 ± 4.5		
Acetoin	513-86-0	3.4	4.9 ± 3.6	0.46	2.7 ± 5.0	>	**
6-Methyl-5-hepten-2-one	110-93-0	2.8	3.5 ± 2.8	0.18	2.5 ± 5.7	>	**
Ethyl acetate	141-78-6	4.8	4.5 ± 2.2	0.59	3.7 ± 9.1	>	**
*cis*-3- Hexenyl acetate	3681-71-8	2.6	3.1 ± 2.4	0	1.0 ± 2.0	>	**
Benzyl acetoacetate	5396-89-4	1.4	1.9 ± 1.5	0.28	1.7 ± 3.9	>	*
Butylated hydroxytoluene	128-37-0	1.7	2.5 ± 1.9	0.01	1.3 ± 4.2	>	**
Benzaldehyde	100-52-7	2.8	3.6 ± 2.9	0.10	0.24 ± 0.48	>	**
Phenol	108-95-2	0.12	0.36 ± 0.78	0.13	1.4 ± 2.3		
**Toluene**	108-88-3	**0.04**	**0.18 ± 0.62**	**3.0**	**7.2 ± 10**	** < **	**
Ethylbenzene	100-41-4	0.04	0.33 ± 0.85	0.17	0.50 ± 1.3		
***m,p*-Xylene**	**106-42-3,108-38-3**	**0.02**	**0.26 ± 1.0**	**0.42**	**1.1 ± 2.1**	** < **	**
o-Xylene	95-47-6	0.03	0.19 ± 0.64	0.05	0.59 ± 1.3		
Styrene	100-42-5	0.02	0.27 ± 1.0	0	0.51 ± 2.0		
p-Dichlorobenzene	106-46-7	0.02	0.30 ± 1.0	0	0.23 ± 0.61		
Geosmin	19700-21-1	0.11	0.58 ± 1.0	0.02	0.48 ± 0.90		
Indole	120-72-9	0.23	0.32 ± 0.37	0	0.13 ± 0.30	>	**
Skatole	83-34-1	0.17	0.32 ± 0.60	0	0.10 ± 0.18	>	**
α-Pinene	2437-95-8	2.5	2.7 ± 1.9	0.65	2.2 ± 5.5	>	**
β-Pinene	127-91-3	2.0	2.6 ± 1.9	0.05	0.59 ± 1.1	>	**
d-Limonene	5989-27-5	2.5	3.3 ± 3.1	0.14	0.39 ± 0.45	>	**
**Methyl mercaptan**	**74-93-1**	**1.4**	**1.8 ± 2.4**	**0.08**	**7.0 ± 20**	** < **	**
**Ethyl mercaptan**	**75-08-1**	**0.53**	**0.77 ± 1.0**	**0**	**4.0 ± 14**	** < **	**
**Allyl methyl sulfide**	**10152-76-8**	**0.35**	**0.71 ± 1.1**	**0**	**1.4 ± 5.8**	** < **	**
Diallyl dimethyl sulfide	2179-57-9	0.24	1.0 ± 2.0	0.18	0.75 ± 1.4		
γ-Hexalactone	695-06-7	1.0	2.9 ± 3.3	0.14	0.74 ± 1.8	>	**
γ-Heptalactone	105-21-5	1.9	3.2 ± 2.8	0.05	0.43 ± 0.69	>	**
γ-Octalactone	104-50-7	1.9	2.5 ± 2.1	0	0.31 ± 0.66	>	**
γ-Nonalactone	104-61-0	1.4	2.7 ± 3.0	0	0.41 ± 1.2	>	**
γ-Decalactone	706-14-9	1.1	2.1 ± 2.5	0.04	0.22 ± 0.36	>	**
γ-Undecalactone	104-67-6	1.3	1.8 ± 1.7	0.02	0.31 ± 0.51	>	**

Skin gases with a greater emission in the PATM group are highlighted in bold. *p < 0.01, **p < 0.001.

### Greater skin gas emissions in the PATM group

Among the 75 skin gases, significantly greater emission fluxes in the PATM group were observed for 2-ethyl-1-hexanol (2E1H), isovaleraldehyde, hexanal, acetone, toluene, *m,p*-xylene, methyl mercaptan, ethyl mercaptan, and allyl methyl sulphide (AMS), most of which potentially have offensive odours and/or lead to adverse health effects owing to exposure.

The emissions of petrochemicals such as 2E1H, toluene, and *m,p*-xylene should be noted because they have been recognised as a chemical factor responsible for idiopathic chemical intolerance^17,18^. Exposure to such chemicals in indoor air, even at ppb levels, is thought to trigger symptoms such as irritation in the skin, eyes, nose, and throat and psychoneurotic symptoms, such as dizziness, nausea, and headache^17,18^. 2E1H is used as a fragrance ingredient and raw material for the production of the plasticiser di(2-ethylhexyl) phthalate^17^. The dermal emission of 2E1H was observed in the left hand of healthy volunteers exposed to 2E1H and/or di(2-ethylhexyl) phthalate vapour in a chemical laboratory^19^. Toluene dermal emissions have also been reported in tobacco smokers^12^. When active smokers smoked a single cigarette, within 15 min, toluene was detected in skin samples collected immediately after the smoking event, together with numerous tobacco-specific chemicals, including nicotine, 3-methylfuran, 2,5-dimethylfuran, and 3-ethenylpyridine^12^. Second-hand smoke also causes dermal toluene emissions. Skin toluene is also emitted from non-smokers when exposed to toluene vapour in a chemical laboratory, where toluene is routinely used as a solvent^19,20^. Therefore, cutaneous emissions of these chemicals can be observed even in healthy subjects when they are exposed to them in their daily lives. However, the emission amounts of these chemicals in the PATM group were considerably higher than those in the non-PATM group: approximately 12 times for 2E1H, 39 times for toluene, and four times for *m,p*-xylene on average. Therefore, we must carefully consider the possibility that the chemicals emitted by the PATM group may induce chemical intolerance in those around them.

The emission of volatile organosulfur compounds such as methyl mercaptan, ethyl mercaptan, and AMS which can cause unpleasant body odour must also be considered. Mercaptans have a malodorous smell such as that of rotten cabbage^21^. AMS is a metabolite of alliin that is present in garlic and is responsible for garlic odour in the breath^22^ and body^23^. Even when garlic is not consumed, skin AMS is observed in healthy volunteers owing to the usual ingestion of various sulphur-containing foods^23^. Because such volatile organosulfur compounds have extremely low odour thresholds of 0.07 ppb for methyl mercaptan, 0.0087 ppb for ethyl mercaptan, and 0.14 ppb for AMS, the increased dermal emissions of sulphur-containing volatiles may change the olfactory impression of body odour in PATM subjects.

Isovaleraldehyde, also known as 3-methylbutanal, has been shown in vitro to be formed by the interaction between human leucocyte antigens and dermal microflora and is suggested to contribute to body odour^24^. Because it has a pungent fruit-like odour, greater emissions may also change the olfactory impression of body odour. Acetone is a product of the metabolic reaction of fatty acids and is necessary to produce energy in the presence of low concentrations of carbohydrates (glucose) in the blood. The ketones in the blood capillaries emerge as a component of sweat and/or rise directly from the blood capillaries to the skin because of its high volatility^10^. The greater emission of skin acetone suggests that the PATM group might include people with eating disorders, a type of mental disorder, because acetone formation is influenced by the period of fasting, starvation, or even the type of diet^25^.

### Skin gases with lower emissions in the PATM group

Significantly lower dermal emissions in the PATM group were observed for lower alcohols (1-pentanol, 1-hexanol, 1-heptanol, 1-octanol, 1-nonanol, and 1-decanol), aldehydes (heptanal, 2-hexenal and benzaldehyde), acetic acid, ketones (2-heptanone, 2-octanone, 2-nonanone, 2-decanone, 2-undecanone, 2-dodecanone, 2-tridecanone, 2-tetradecanone, 2-pentadecanone, acetoin (3-hydroxybutan-2-one), and 6-methyl-5-hepten-2-one), acetates (ethyl acetate, cis-3-hexenyl acetate, and benzyl acetate), indole, skatole (3-methylindole), α-pinene, β-pinene and d-limonene, and volatile cyclic esters (γ-hexalactone, γ-heptalactone, γ-octalactone, γ-nonalactone, γ-decalactone, and γ-undecalactone). To the best of our knowledge, it is difficult to explain the lower emissions of each gas in the PATM group. However, several of them provide an aroma with a comfortable impression; thus, they are also utilised as flavours or fragrances. Woody smell is known to act as a mood relaxant^26^, and α-pinene, β-pinene, and d-limonene are typical aromatic components of wood or wood-derived interior materials. Inhalation of d-limonene has been shown to enhance parasympathetic nervous activity, decrease heart rate, and provide ‘comfortable’ stimulation^27^. The essential oils containing these aroma compounds are widely used in aroma therapy, a holistic healing treatment that uses natural plant extracts to promote health and well-being. γ-lactones are volatile cyclic esters naturally present in fruits, including peach, plum, apricot, pineapple, and strawberry^28,29^. They contribute to the sweet smell of fruits and sweet body scents, particularly in teen girls^30^. The loss of such skin aroma components may also affect the olfactory impression of body odour in subjects with PATM.

Acetic acid is one of the simplest carboxylic acids and its vapour has a pungent vinegar-like odour. It is produced by the bacterial degradation of precursors such as leucine and isoleucine in sweat and is associated with body odour in young adults^31^. Dermal emissions of skin acetic acid have been shown to increase with an increase in the amount of sweat during exercise. Therefore, skin acetic acid levels may be a good indicator of perspiration^32^. Although sweating is considered a key factor affecting human body odour, the lower dermal emission flux in subjects with PATM indicates that neither physical nor psychological sweating is the cause of the characteristic skin gas profile of people who complain of PATM.

**Figure 1:**
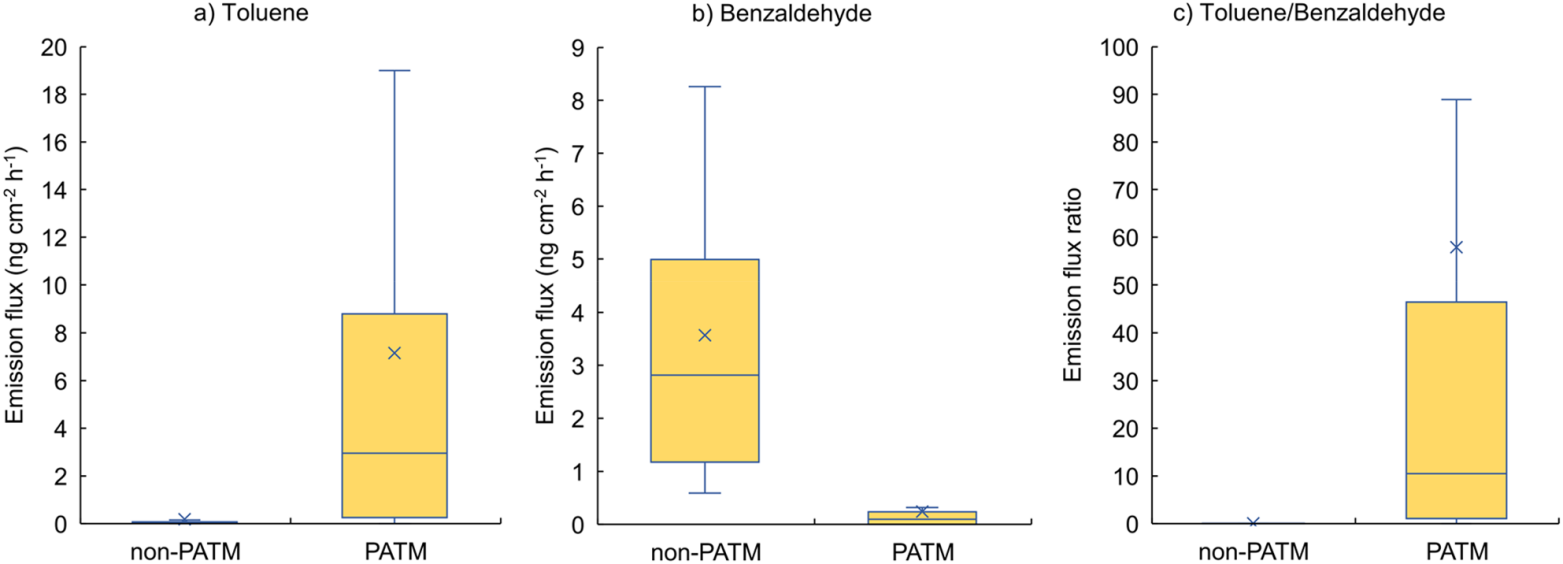
Box-and-whisker plots of dermal emission fluxes of toluene, its possible metabolite benzaldehyde and ratio of toluene and benzaldehyde for non-PATM and PATM groups. Mark x denotes the mean values.

Among the aldehydes with lower dermal emissions in PATM group, we focused on benzaldehyde as a possible metabolite of toluene^20^. Figure 1 shows a comparison of the dermal emission fluxes of toluene and benzaldehyde and the ratio of toluene to benzaldehyde between non-PATM and PATM subjects. Significantly greater emissions were observed for skin toluene in the PATM group (*p* ≤ 0.0001) with approximately 39 times those of non-PATM subjects on average, whereas significantly lower emissions were observed for skin benzaldehyde in the PATM group (*p* ≤ 0.0001). The mean ratio of toluene to benzaldehyde was 58 in patients with PATM and much greater than 0.076 in non-PATM subjects. Therefore, the toluene-to-benzaldehyde ratio is considered a vital sign that suggest the fundamental of PATM.

### Comparison with odour threshold value

**Figure 2:**
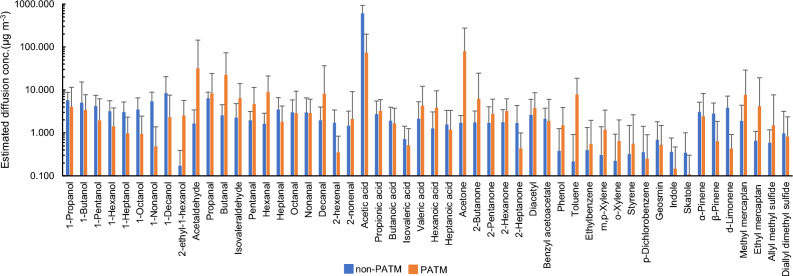
Estimated diffusion concentrations of 52 skin gases released from non-PATM and PATM subjects at a distance of 50 cm from a non-PATM or PATM emitter in a modelled room (volume: 32 m^3^, air change rate: 0.5 h^−1^). Bar graphs show the mean diffusion concentrations of each skin gas and error bars show the standard deviations.

The PFS methodology determines the dermal emission flux in units of ng cm^−2^ h^−1^ which cannot be compared with the odour threshold value (OTV) reported in units of ppb or µg m^−3^. Thus, using the dermal emission flux data shown in Table 1, we estimated the indoor air concentrations of skin gases diffused from an emitter using a two-component box model^33^ and compared them with the OTVs of 52 skin gases previously reported^34^. Figure 2 shows the estimated diffusion concentrations of skin gases released from non-PATM and PATM subjects, assuming the single emitter stayed in a room (volume: 32 m^3^, air change rate: 0.5 h^−1^, a typical living room of a Japanese house) and another neighbouring person was exposed to the diffused skin gases at 0.50 m from the emitter. Bar graphs show the mean diffusion concentrations of each skin gas and error bars show the standard deviations. The mean diffusion concentration ranged from 0.17 µg m^−3^ (2E1H) to 6.1 × 10^2^ µg m^−3^ (acetic acid) in the case of non-PATM emitters and from 0.11 µg m^−3^ (skatole) to 79 µg m^−3^ (acetic acid) in PATM emitter under the environmental condition.

**Figure 3:**
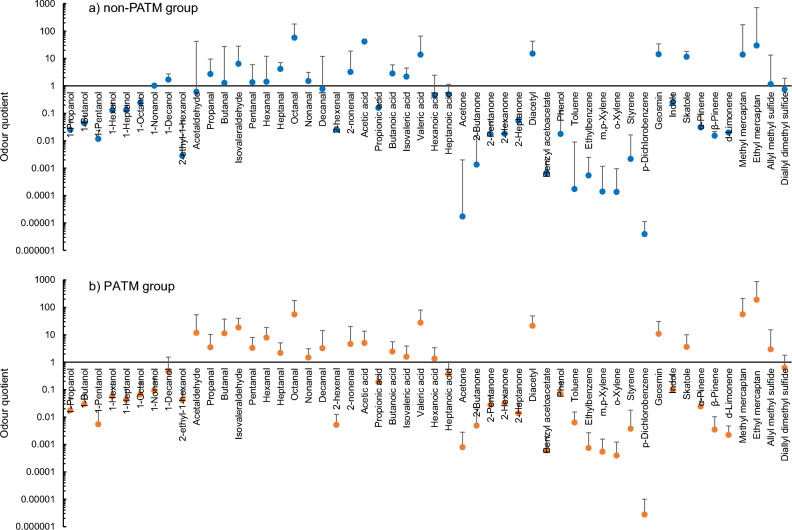
Odour quotients of 52 skin gases for non-PATM group(a) and PATM group (b) at a distance of 50 cm from an emitter in a modelled room (volume: 32 m^3^, air change rate: 0.5 h^−1^) using the estimated diffusion concentrations shown in Fig. 2. Plots show mean OQ values with positive standard deviations for easy comparison with unity (OQ = 1).

To determine the relative odour strength of the skin gases, the odour quotient (OQ), which is the ratio of the diffusion concentration to the OTV of each gas^35^, was calculated. If the OQ is greater than unity, the skin gas can contribute to the body odour or scent of the emitter which can be sniffed by a neighbouring person. Figure 3 shows the results. The mean OQ values were plotted with positive standard deviations for easy comparison with unity. As shown in Fig. 3a, a relatively high OQ was observed for non-PATM emitters for isovaleraldehyde, octanal, acetic acid, valeric acid, diacetyl, geosmin ((4*S*,4a*S*,8a*R)*-4,8a-Dimethyloctahydronaphthalen-4a(2*H*)-ol), skatole, methyl mercaptan, and ethyl mercaptan. In addition to these components, aldehydes such as acetaldehyde, butanal, hexanal, and AMS were found to be associated with the body odour of PATM emitters with OQs greater than unity, as shown in Fig. 3b. The sum of mean OQs of 52 skin gases for the PATM emitters was calculated to be 457, which was approximately double that for the non-PATM emitters (SOQ = 232). The actual odour strength and quality are determined by the olfactory system that receives the mixed gases. However, this estimation is effective in revealing the gases that have a potential effect on the olfactory impression of the body odour of patients with PATM.

Although greater cutaneous emissions of 2E1H, toluene, and *m,p*-xylene were observed in the PATM group, these petrochemicals did not seem to contribute to the body odour of the PATM subjects because of their extremely low OQs, such as 0.042 for 2E1H, 0.0063 for toluene, and 0.0053 for *m,p*-xylene. Furthermore, notably, the diffusion concentration of toluene was estimated to be 7.8 µg m^−3^ in the case of PATM emitters and was much lower than the indoor air quality guideline of Japan for toluene (260 µg m^−3^). In addition, 1.2 µg m^−3^ of the diffusion concentration of *m,p*-xylene was much lower than the guideline value of 200 µg m^−3^.

## Discussion

It is currently difficult to propose a mechanism to explain the significant differences in human skin gas profiles between the groups because the occupations, medical histories, life behaviours, and living environments of the subjects are diverse. To date, no sex differences or age dependence have been observed. However, a careful reading of the vital signs shown in Table 1 may provide a hint for solving this complex issue.

One possible key component is toluene and its metabolite benzaldehyde, as measured in this study. To the best of our knowledge, there are no reports on toluene synthesis in humans in vivo. Therefore, skin toluene is most inhaled and is released via the blood route, as previously reported^20^. Usually, a greater portion of inhaled toluene is metabolised to hippuric acid in a cascading manner and then excreted in the urine^36^. The initial step in toluene metabolism is side-chain hydroxylation to benzyl alcohol, catalysed predominantly by the hepatic cytochrome P450 (CYPs) superfamily^37^. These five CYPs are involved in toluene metabolism: CYP1A2, CYP2B6, CYP2E1, CYP2C8, and CYP1A1^38^. Benzyl alcohol is converted to benzaldehyde by alcohol dehydrogenase (ADH) and subsequently metabolised to benzoic acid by aldehyde dehydrogenase (ALDH). Benzoic acid is conjugated with glycine and eliminated from the urine as hippuric acid. Therefore, benzaldehyde is a volatile intermediate in toluene metabolism. When the activity of CYPs related to the toluene mechanism was inhibited, the dermal emission of unchanged toluene increased and that of benzaldehyde decreased, resulting in a greater ratio of toluene to benzaldehyde as shown in Fig. 1. Therefore, the toluene-to-benzaldehyde ratio can be a good indicator of diagnosing PATM or not being PATM.

Other possible key components were hexanal and octanal. Octanal is an aldehyde that is a product of skin lipid oxidation and has a fruit-like odour^39^. Skin octanal was suggested as one of the ingredients that affect human body odour the most, as shown in Fig. 1b, and is usually observed in the indoor air of residential houses^40^. Moreover, hexanal is a lipid oxidation product. However, it is also considered an oxidative stress marker in exhaled breath with an unpleasant hay-like odour^41^. Oxidative stress is defined as a disturbance in the balance between the cellular production of reactive oxygen species and antioxidant defences^42^. The brain, which has high oxygen consumption and a lipid-rich environment, is considered highly susceptible to oxidative stress^42^ that is implicated in several mental disorders, including depression, anxiety disorder, bipolar disorder, eating disorder, schizophrenia, and body dysmorphic disorder, some of which are often observed in people with PATM with increasing risk of suicide. Willems et al.^43^ have reported the dermal emission flux of hexanal was significantly reduced by the intake of New Zealand Black Currant powder probably because of the antioxidant and anti-inflammatory effects of flavonoid anthocyanins abundant in the fruit^44^. This suggests that in vivo oxidative species contribute to lipid oxidation, resulting in a greater emission of skin hexanal in patients with PATM.

Autodysomophobia or olfactory reference syndrome (ORS) has long been known as a symptom related to body odour in psychiatry. This is a preoccupation with the false belief that one is emitting a foul or offensive body odour^45^. PATM seems to differ from autodysomyophobia or ORS in that it affects the people around them, at least based on descriptions by people with PATM. Greater emissions of gases were observed in the PATM groups with potential offensive odours and/or adverse health effects, as described above, and there are people who are very vulnerable to low doses of chemicals in the environment. To better understand PATM, we must further investigate the people affected by exposure to human skin gases released by people who complain of PATM.

## Conclusion

The skin gas profiles of people who claimed to have PATM were investigated by measuring the dermal emission fluxes of 75 skin gases using PFS and GC/MS. Although the PATM and non-PATM groups were assigned simply based on their declaration, there was a significant difference in the cutaneous emission of several gases between the two groups: greater emissions of petrochemicals, organosulfur compounds, and some aldehydes and lower emissions of aroma compounds and others. No sex differences or age dependences were observed. It is currently difficult to propose a mechanism to explain the characteristic skin gas profile in the PATM group. However, the ratio of toluene to its metabolite benzaldehyde is considered a vital sign that suggests the fundamental of PATM in association with an activity of hepatic CYPs. The greater emission of hexanal, a possible oxidative stress marker, may be another vital sign due to its contribution to olfactory impression of body odour of people who claimed PATM. These findings indicate that PATM is worthy of further research and requires an interdisciplinary approach as a phenomenon or symptom that has not yet been medically elucidated.

## Methods

### Measurement of human skin gases

**Figure 4:**
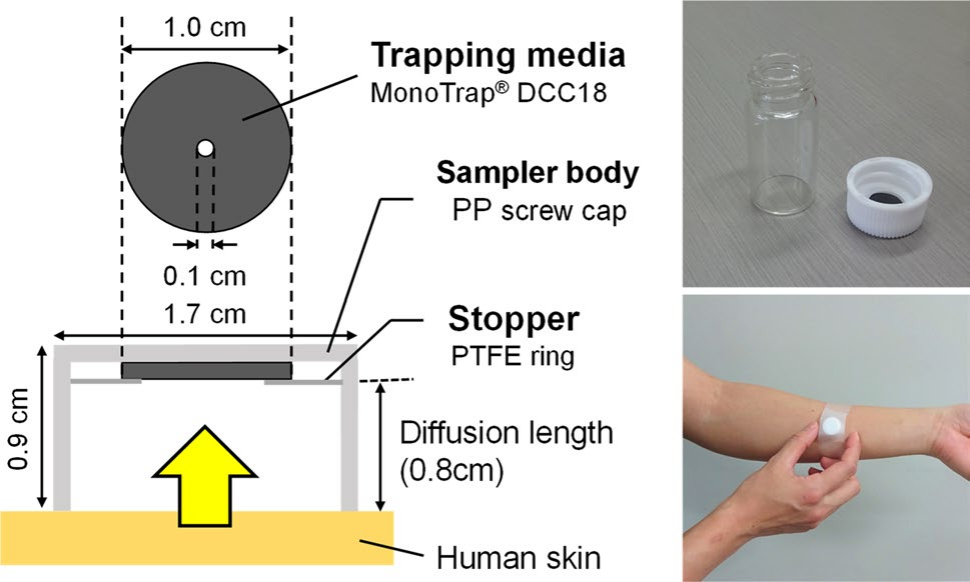
Schematic view of the PFS for the measurement of 75 skin gases used in this study.

Japanese participants were voluntarily recruited through SNS and word-of-mouth. Volunteers working in facilities that used chemicals were excluded. The subjects were assigned to two groups, non-PATM and PATM, based on whether they claimed they were suffering from PATM. The participants were asked to collect their own human skin gas from the non-dominant forearm by the PFS (MonoTrap^®^ SG DCC18, GL Sciences, Tokyo, Japan)^10,11^ at any time for 1 h in their house, school, or workplace without any limitation for their act before and during the samplings. Figure 4 shows a schematic of the PFS. The participants were allowed to use their dominant arms during sampling. No special treatment was performed on the forearm surface before sampling. The PFS was anchored on the skin surface by a piece of surgical tape (approx. 12 cm length × 2.5 cm width, Yu-ki Ban^®^, Nitto, Tokyo, Japan). After sampling, the PFS was sent to the Tokai University laboratory via a home delivery service.

The collected skin gases were analysed using gas chromatography/mass spectrometry as described in previous works^10,11,23,43^. In brief, the trapped skin gases were eluted into 500 µL of dichloromethane with 15 min of ultrasonic extraction. The extracts were analysed using a gas chromatograph model 7890B (Agilent Technologies, Santa Clara, CA, USA) and a mass spectrometer (JMS-Q1050GC MkII, JEOL, Tokyo, Japan). One microlitre of sample extracts, blank extracts, and quantitation standards was injected with a split ratio of 30:1 into a InertCap WAX-HT capillary column (30 m × 0.25 mm I.D., 0.25 µm film thickness, GL Science, Tokyo, Japan). The carrier gas was helium (G1 grade, Taiyo Nippon Sanso, Tokyo, Japan) at a flow rate of 1.0 mL min^−1^. The injector port was maintained at 260 °C. The oven temperature was programmed as follows: held at 40 °C for 5 min, ramped at 8 °C·min^−1^ to 150 °C, and ramped at 16 °C·min^−1^ to 260 °C and held for 6 min. Sample extract signals were acquired using the real-time selected ion monitoring (SIM) mode. The target analytes were 75 skin gases (Table 1). The emission flux of the human skin gas, *E* (ng cm^−2^ h^−1^), was obtained using Eq. (1):

1 $$E = W/\left( {S \cdot t} \right)$$

where *W* is the amount (ng) of collected human skin gas, *S* is the effective cross-section of the adsorbent (0.594 cm^2^), and *t* is the sampling duration (1.0 h).

### Assessment of odour quotient

**Figure 5:**
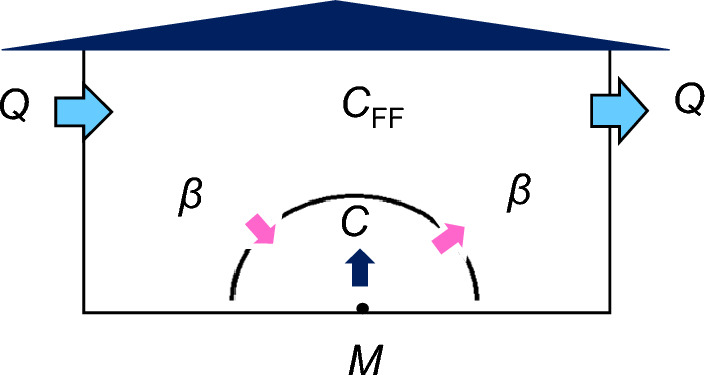
Schematic drawing of the two two-component box model for the estimation of diffusion concentrations of skin gases released from the whole body of non-PATM and PATM emitters assuming the emitter as a point source.

To assess the potential exposure to human skin gases released from an emitter in a room, a two-component box model, near-field and far-field model^33^, was used to estimate the diffusion concentration of skin volatiles. The region near and around the emitter was modelled as a well-mixed box (near field), and the rest of the room was modelled as another well-mixed box (far field). A certain amount of air was exchanged between the boxes. A schematic of these two boxes is shown in Fig. 5. Assuming a steady-state condition, the indoor air concentration of skin gases diffusing from the emitter to the near field, *C* (g m^−3^), was calculated using Eq. (2).

2 $$C = M/Q + M/\beta$$

*M* is the emission rate of skin gas from an emitter (g h^−1^) which can be obtained by multiplying the dermal emission flux (g cm^−2^ h^−1^) and the whole-body surface area (approximately 16,000 cm^2^ for Japanese people). The air change rate *Q* was set at 16 m^3^ h^−1^ assuming a typical room of a Japanese house with a 32 m^3^ inner volume and 0.5 h^−1^ air change rate. The term* β* is an exchange rate between two boxes and is a function of the distance between the emitter and a neighbouring person (a radius of the hemisphere of a near-field box), *r*(m), as shown in Eq. (3):

3 $$\beta = \pi r^{2} v$$

where *v* is an air flow (m h^−1^) in a room. The diffusion concentrations at a distance of 0.5 m apart from an emitter were calculated using the average dermal emission flues of each skin gas in the non-PATM and PATM groups shown in Table 1. The airflow was set at 0.06 m s^−1^ (= 216 m h^−1^).

The OQ was calculated for 52 skin gases using the estimated diffusion concentration at 0.5 m and the odour threshold value, *OT* (g m^−3^), converted from the reported values (ppb) at 298 K.

4 $$OQ = C/OT$$

### Statistical analysis

Statistical analyses were performed on JMP^®^14.2 for windows. Differences in dermal emission fluxes between the non-PATM and PATM groups were analysed using the Wilcoxon signed-rank test. Statistical significance was set at *p < 0.01 and **p < 0.001.

### Ethics approval and consent to participate

This study was performed in accordance with the guidelines laid out in the Declaration of Helsinki and was conducted with the approval of the Institutional Review Board, Shonan Campus, Tokai University, Japan (No. 16181, 18063, 19057, 21041). Written informed consent was obtained from all participants.

## Data Availability

All data generated or analysed during this study are included in this published article.
